# Size Matters: Biological and Food Safety Relevance of Leaf Damage for Colonization of *Escherichia coli* O157:H7 *gfp*+

**DOI:** 10.3389/fmicb.2020.608086

**Published:** 2021-01-27

**Authors:** Emina Mulaosmanovic, Sofia T. Windstam, Ivar Vågsholm, Beatrix W. Alsanius

**Affiliations:** ^1^Microbial Horticulture Unit, Department of Biosystems and Technology, Swedish University of Agricultural Sciences, Alnarp, Sweden; ^2^Bacteriology and Food Safety Unit, Department of Biomedical Sciences and Veterinary Public Health, Swedish University of Agricultural Sciences, Uppsala, Sweden

**Keywords:** enterohemorrhagic *E. coli*, food safety, internalization, leafy vegetables, lesions, risk assessment, shiga-toxigenic *E. coli*, spinach (*Spinacia oleracea L*.)

## Abstract

This study examined the biological and food safety relevance of leaf lesions for potential invasion of food pathogens into the plant tissue (internalization). This was done by determining the role of artificial leaf damage in terms of damaged leaf area on proliferation of *E. coli* O157:H7 *gfp*+. In a two-factorial experiment, unwashed fresh baby leaf spinach (*Spinacia oleracea* L.) was subjected to four damage levels (undamaged, low, moderate, high damage; factor 1) and three incubation intervals (0, 1, 2 days post-inoculation; factor 2). Individual leaves were immersed for 15 s in a suspension loaded with *E. coli* O157:H7 *gfp*+ (10^6^ CFU × mL^–1^). The leaves were analyzed individually using image analysis tools to quantify leaf area and number and size of lesions, and using confocal laser scanning and scanning electron microscopy to visualize leaf lesions and presence of the introduced *E. coli* strain on and within the leaf tissue. Prevalence of *E. coli* O157:H7 *gfp*+ was assessed using a culture-dependent technique. The results showed that size of individual lesions and damaged leaf area affected depth of invasion into plant tissue, dispersal to adjacent areas, and number of culturable *E. coli* O157:H7 *gfp*+ directly after inoculation. Differences in numbers of the inoculant retrieved from leaf macerate evened out from 2 days post-inoculation, indicating rapid proliferation during the first day post-inoculation. Leaf weight was a crucial factor, as lighter spinach leaves (most likely younger leaves) were more prone to harbor *E. coli* O157:H7 *gfp*+, irrespective of damage level. At the high inoculum density used, the risk of consumers’ infection was almost 100%, irrespective of incubation duration or damage level. Even macroscopically intact leaves showed a high risk for infection. These results suggest that the risk to consumers is correlated with how early in the food chain the leaves are contaminated, and the degree of leaf damage. These findings should be taken into account in different steps of leafy green processing. Further attention should be paid to the fate of viable, but non-culturable, shiga-toxigenic *E. coli* on and in ready-to-eat leafy vegetables.

## Introduction

Consumers, trade, and producers have been repeatedly alerted to the risk of food illness outbreaks related to leafy green vegetables (Mogren et al., 2018). Amongst causal agents, shiga-toxigenic *Escherichia coli* is of particular interest due to its very low infectious dose (EFSA Panel on Biological Hazards (BIOHAZ), 2013) and impact on public health. Irrigation water, organic manure, soil, domestic and wild animals, and poor hand hygiene and health among processing workers have been listed as main transmission routes (Fan et al., 2009).

Several studies have shown the potential for internalization of foodborne pathogens inside leaves (Brandl, 2008; Holden et al., 2009; Deering et al., 2012; Erickson, 2012; Hirneisen et al., 2012; Wright et al., 2013; Merget et al., 2019). Internalization can occur under conditions mimicking field production (Solomon et al., 2002; Mitra et al., 2009), in processing steps involving bruising, chopping, or slicing, and during packing (Van der Linden et al., 2016). However, there is an ongoing debate on whether internalization is a laboratory artifact or a natural phenomenon (Warriner et al., 2003; Hora et al., 2005; Zhang et al., 2009; Erickson et al., 2010, 2014; Macarisin et al., 2014; Hartmann et al., 2017). In this debate, the “intact” leaf is the common basis for discussion, ignoring the fact that an intact leaf, without any wounds, is not found outside the laboratory (Mulaosmanovic et al., 2020). Plant lesions are localized areas of damaged and dead cells on plant surfaces, and they are characteristic of the plant sessile lifestyle. Lesions can have biotic or abiotic origins, e.g., hail, wind, soil, machines, herbivore grazing, and pest and pathogen damage. In nature and primary plant production, leaf damage is the norm. Injuries are irreversible and lead to cellular and tissue ruptures within the plant, and in more severe form to organ breaking (Luengo et al., 2008). As baby leaf tissue is sensitive, disruption or breaking of the epidermal layer can occur from primary production to consumer. Upon wounding, although unable to close cuticle and epidermis, plants form scar tissue from suberized cells (Kolattukudy, 1981). Once injured, the integrity of the epidermis is compromised and solutes leach onto the leaf surface. This perturbation alleviates local resource scarcity and provides nutrients that can support prolonged survival of microorganisms (Aruscavage et al., 2008) and successful colonization by invaders (Mallon et al., 2015), making injured sites preferred microbial habitats (Brandl, 2008). Pathogens such as *E. coli* O157:H7 that lack enzymes for degrading plant cell wall components often utilize damaged sites for attachment (Han et al., 2000) and invasion of intact leaf tissues (Deering et al., 2012; Savatin et al., 2014). This provides protection from environmental stresses encountered in the otherwise hostile phyllosphere (Brandl and Mandrell, 2002).

Mulaosmanovic et al. (2020) presented tools for visualizing micro-, meso- and macrolesions on leafy vegetables and confirmed the presence of lesions on leaves macroscopically assumed to be intact. By following field-grown leafy vegetables from a commercial site through the processing chain, Mulaosmanovic et al. [submitted] demonstrated that the level of leaf damage increases from farm to fork. However, from the current literature, it is not clear whether lesion size and total lesion area contribute to the probability of shiga-toxigenic *E. coli* adhering to leaves. Many previous studies have examined *E. coli* adhesion (Saldaña et al., 2011; Macarisin et al., 2012, 2013; Rossez et al., 2014), and persistence (Patel et al., 2010; Choi et al., 2011) on leaves, but have rarely or never considered lesions in parallel. While other studies have assessed impact of damage on *E. coli* growth (Aruscavage et al., 2008; Brandl, 2008; Hartmann et al., 2017), the role of different damage levels for the proliferation of *E. coli* O157:H7 were not previously evaluated. To our knowledge, this is the first study where the biological and food safety relevance of relative leaf damage and size of individual lesions for *E. coli* O157:H7 establishment was evaluated on leaf scale. The findings from this study are of interest for the establishment of hurdles and measures preventing transmission within the horticultural value network.

The main research questions considered in the present study were: “Is leaf damage level of biological relevance for the adhesion and internalization of O157:H7 *gfp*+?” and if so, “Are there concomitant food safety consequences?”. Non-washed, macroscopically “intact” baby spinach leaves obtained from a commercial processing company were subjected to three levels of artificial damage before dip inoculation with *E. coli* O157:H7 *gfp*+, reflecting modifications to two fundamental environmental factors, namely nutrient availability/pulse and site. The hypotheses tested were:

(i) Adhesion of *E. coli* O157:H7 *gfp*+ on spinach leaf surface is enhanced by increasing lesion area.
(ii) Any size of leaf wound enables *E. coli* O157:H7 *gfp*+ to internalize into spinach leaf.
(iii) The number of internalized *E. coli* O157:H7 *gfp*+ cells increases with increasing wound size and relative leaf damage.

## Materials and Methods

### Plant Material and Artificial Damage

Spinach (*Spinacia oleracea* L.) grown in Italy under conventional farming practices and harvested at baby leaf stage (BBCH stage 13) was used in the experiments (January–May 2020). The spinach was imported by the Swedish company Vidinge Grönt AB (Kävlinge, Sweden) as unwashed material. Leaves that showed no visible symptoms of damage were selected for the experiments.

**FIGURE 1:**
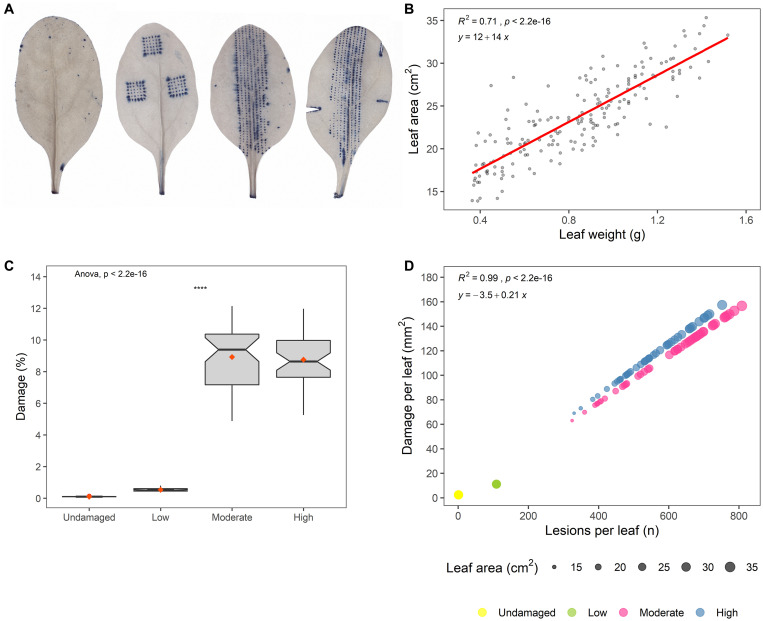
**(A)** Images of artificially damaged and Trypan blue-stained spinach leaves, visualizing the different levels of standardized artificial damage (from an undamaged leaf on the left to a high-damage leaf on the right). This batch of artificially damaged leaves was used for predictions of damage and number of lesions on *E. coli* O157:H7 inoculated leaves. **(B)** Plot of leaf weight (g) against leaf area (cm^2^), with regression line (red) showing the result of the model: *y*_*Leaf area*_ = β_0_+β_1_*x*_*Leaf weight*_. **(C)** Predicted damage per leaf (%) on *E. coli* O157:H7 inoculated leaves (Wilcoxon test; ns: *p* > 0.05, *p ≤ 0.05, **p ≤ 0.01, ***p ≤ 0.001, ****p ≤ 0.0001). **(D)** Predicted number of leaf lesions plotted against damaged leaf area (mm^2^) for different damage levels, where point size in scatter plots denotes leaf area of *E. coli* O157:H7 inoculated leaves.

Different levels of standardized artificial damage in known patterns were inflicted upon the spinach leaves using a Derma stamp (HudRoller Of Sweden; 36 microneedles; 1 mm) or a Derma roller (HudRoller Of Sweden; 1 mm). Both of these generate dot-like lesions. Additionally, a scalpel was used to inflict more severe damage to leaves (Figure 1A). The extent of artificial damage was varied to generate four different levels of damage severity: undamaged (U), low (L), moderate (M), and high (H).

Leaves in the undamaged (U) group were not artificially damaged, and only damage to the leaf petiole caused by harvest was considered during damage quantification. Although this treatment served as a control, it did not preclude presence of microscopic damage, as leaves used in all treatments were only macroscopically observed from the abaxial and adaxial sides.

For low-level (L) damage, a total of 108 lesions of the same size were inflicted using the Derma stamp tool (3 × 36), causing total of about 0.1 cm^2^ damage per leaf. Damage at level L was constant and independent of leaf size, and thus smaller leaves had higher relative damage than large leaves.

In moderate-level (M) damage, a Derma roller tool was used to inflict lesions along the midrib area, with about 0.04 cm^2^ damage per cm^2^ leaf area. The amount of damage was not constant, as the number of individual lesions per leaf varied with leaf area (small leaves had fewer lesions than large leaves), but relative damage per leaf was constant and not affected by leaf size. Individual lesions inflicted at damage level M were of same size, and were more numerous than at damage level L, but smaller in size.

For high-level (H) damage, leaves were first damaged as in group M and then damaged further by making three cuts with a scalpel, giving cut-related damage that can potentially occur during harvest and post-harvest processing (Mulaosmanovic et al., submitted). At damage level H, about 0.05 cm^2^ damage was inflicted per cm^2^ of leaf area. Number of lesions was not constant, but relative damage per leaf was not affected by leaf size.

As both downstream damage quantification and microorganism extraction methods were destructive, one separate batch of artificially damaged leaves was prepared per method. Measured leaf and lesion areas obtained from batch used for damage quantification were further used as a basis for prediction of leaf-scale damaged area (mm^2^, %) and number of lesions on *E. coli* O157:H7 inoculated leaves.

### Bacterial Strain, Inoculation, and Growth Conditions

An *E. coli* O157:H7 strain, lacking virulence factors verotoxin-1 and -2, but expressing *eae*, was used for the experimental work. This strain hosts the pGLO plasmid, which encodes for ampicillin resistance and green fluorescent protein (GFP), coupled with an arabinose responsive promoter. The bacteria were routinely grown in lysogeny broth (LB; Sigma-Aldrich, United States) supplemented with ampicillin (100 μg mL^–1^; Sigma-Aldrich, Belgium) and arabinose (0.2%; Merck KGaA, Germany) for approximately 18 h at 37°C. Details of the *E. coli* O157:H7 inoculum preparation protocol are provided by El-Mogy and Alsanius (2012). The final concentration of inoculum solution was set to 10^6^ CFU of *E. coli* O157:H7 *gfp*+ × mL^–1^ in 0.085% NaCl.

Directly post-damage, spinach leaves used for all damage levels (*n* = 15) were individually dip-inoculated by immersing them for 15 s in the inoculum suspension using forceps. The leaf petiole was kept outside the inoculum during inoculation, to prevent contact of the inoculant with harvest damage to the basal edge of the petiole. Excess inoculum suspension was removed by holding the leaf tip against a paper towel until all excess suspension had been removed.

*E. coli* O157:H7 *gfp*+ was extracted from individual spinach leaves directly post-inoculation for all damage levels (0 days post-inoculation, dpi), in order to determine the amount of bacterial cells added to single leaves. The remainder of the inoculated leaves were incubated in sealed plastic bags at room temperature for 24 h (1 dpi) and 48 h (2 dpi) (Supplementary Figure 1 in Supplementary Material).

### Analyses

#### Leaf Area and Lesion Area Measurement Based on Image Analysis

Visualization and quantification of artificially induced damage followed the protocol devised by Mulaosmanovic et al. (2020). In brief, to enhance contrast between healthy and damaged (later stained) tissue of leaves from the batch used for damage quantification, chlorophyll was removed from the leaf tissue by soaking whole detached leaves in a clearing solution composed of ethanol (Solveco, 95%) and acetic acid (Acros Organics, 99.6%) in a 3:1 (v/v) ratio. Leaves were soaked in clearing solution until they became entirely transparent. To visualize leaf tissue damage, cleared leaves were stained with 0.01% Trypan Blue dye (Sigma-Aldrich) in de-ionized water (diH_2_O) for 4 h on a rotary shaker (50 rpm), followed by washing with diH_2_O.

Stained leaves were placed on a LED light table and photographed using a Canon EOS 5D Mark IV camera. A reference standard size object (1 cm^2^) was included in the image for calibrating the dataset. The camera was fitted with a Canon EF 50 mm 1:1.4 lens, and operated in manual exposure mode (shutter speed 1/125, aperture 6.3, ISO 160).

Leaf and lesion areas were then quantified using the LiMu image analysis program (Mulaosmanovic et al., 2020). Absolute damaged area was expressed in pixels (px), as the sum of individual lesion areas per leaf, and later calibrated to mm^2^. Relative damage (%) per leaf was calculated as:

(1) $$D\,a\,m\,a\,g\,e=\left(\frac{L\,e\,s\,i\,o\,n\,a\,r\,e\,a}{L\,e\,a\,f\,a\,r\,e\,a}\right)\times 100$$

Leaf photographs were taken prior to inoculation, in order to quantify the leaf area of inoculated leaves. Images of leaves were captured as described for the stained leaves. The area was measured using the IMAGEJ open-source image analysis software. By doubling the measured leaf area, the adaxial and abaxial surfaces were considered in the total surface area. The double-sided leaf area was used in all area-related calculations.

#### Microbial Analysis

In order to assess *E. coli* O157:H7 *gfp*+ proliferation and internalization rate on leaves with different levels of artificial damage, bacteria were extracted from individual leaves in a series of steps: detachment, washing, surface sterilization, verification of residual viable counts associated with the leaf surface (leaf print), and maceration.

##### Detachment

Epiphytically residing *E. coli* O157:H7 *gfp*+ cells were detached from individual leaves in 50 mL Tris-buffer (0.01M, pH 8, Tris(hydroxymethyl)aminomethane hydrochloride, Merck, Germany) by centrifuging (Eppendorf AG 5804, Germany) at 3000 × *g* for 15 min.

##### Washing

To wash off remaining supernatant on the leaf surface after detachment, leaves were moved to fresh sterile vials with 50 mL Tris buffer and centrifuged at 3000 × *g* for 2 min.

##### Surface sterilization

Individual leaves were immersed in 50 mL freshly prepared sodium hypochlorite (Klorin, 27 g L^–1^, Colgate Palmolive, Sweden) to remove remaining artificially added *E. coli* potentially residing on the leaf surface after the washing step. This corresponds to the concentration used for disinfection of leaves in commercial settings (150 ppm) (Niemira, 2007). Leaves in disinfectant were mildly agitated for 15 min. Disinfectant residues were removed by submersing leaves in a tube containing 50 mL sterile diH_2_O and centrifuging at 3000 × *g* for 2 min, before placing wet leaves on paper towel and allowing them to air-dry.

##### Leaf imprints

Presence of any surface-residing *E. coli* O157:H7 post-surface sterilization was determined by pressing surface-sterilized leaves (both abaxial and adaxial sides) onto LB agar plates (20 g LB Broth, and 15 g of Bacto agar, DIFCO, United States), supplemented with ampicillin (100 μg mL^–1^) and arabinose (0.2%).

##### Maceration

Leaves were weighed, and leaf macerate was generated with sterile scientific precision blown glass macerators (manufactured at the Department of Chemistry, Lund University, Sweden) in Tris buffer. The amount of maceration buffer was adjusted based on leaf fresh weight:

(2) B⁢u⁢f⁢f⁢e⁢r⁢(m⁢L)=W⁢e⁢i⁢g⁢h⁢t⁢(g)×2

To measure the total bacterial counts from the detachment, washing, surface sterilization, and maceration steps, serial dilutions of supernatant or leaf macerate from each sample were plated in triplicate on LBA plates, incubated at 37°C for approximately 18 h, and then enumerated under UV-light (Spectroline EA-160/FE, Spectronics Corporation, NY, United States). Leaf imprint plates were incubated in same manner, and colonies were enumerated.

#### Scanning Electron Microscopy (SEM)

Leaf discs (Ø 8 mm) were extracted from intact or wounded areas on *E. coli* O157:H7-inoculated leaves with a coring tool (Harris Uni-Core) at 2 dpi. The discs were immediately immersed in fixative (2% paraformaldehyde and 2% glutaraldehyde) overnight, and washed in 0.1 M sodium cacodylate buffer, with pH adjusted to 7.3. After fixation, each specimen was dehydrated in a graded series of ethanol (70%, 96%, and 100% twice, for 10 min at each step), followed by drying in a critical point dryer (CPD; Bal-Tec CPD 030), using liquid CO_2_ as transitional fluid and CO_2_ gas to remove all ethanol remaining in samples. Samples were affixed to aluminum stubs with double-sided adhesive tape, and sputter-coated with gold ions (Sputter coater Cressington 108 auto) at 20 mA for 45 s.

Imaging of the adaxial epidermal surface was performed with a high-resolution Scanning Electron Microscope (SEM; HITACHI SU3500) at 5 kV, using a secondary electron (SE) detector and working distance 5–10 mm.

#### Confocal Laser Scanning Microscopy (CLSM)

Leaves used for microscopy observation were surface-sanitized by washing in 0.25% sodium hypochlorite for approximately 20 s (Hartmann et al., 2017). Leaf discs (Ø 8 mm) were punched out with a coring tool from intact or wounded areas on GFP-tagged *E. coli* O157:H7-inoculated leaves at 2 dpi. The discs were placed on glass slides in phosphate-buffered saline solution (0.01 M, pH 7.4), covered with cover slips (1.5H; Carl Zeiss Microscopy) and sealed with paraffin to allow prolonged imaging. The adaxial surface of the leaf, where artificial wounds were inflicted, was observed using a Zeiss LSM880 confocal laser scanning microscope (Carl Zeiss Microscopy) fitted with 40× LD LCI Plan-Apochromat 1.2 water immersion AutoCorr DIC objective. The imaging was focused on the wounded areas and on areas adjacent to wounds. GFP (green) and chlorophyll (red) were excited at 488 and 561 nm, and the emissions were collected using 493–531 and 661–701 nm filters, respectively.

#### Risk Assessment

To characterize risk, a dose-response model was used to estimate the probability of infection, P_inf_, resulting from exposure to consumption of a single serving with a certain pathogen dose *d*. The model considered only *E. coli* O157:H7 *gfp*+ re-isolated from the macerate. Following a review of dose-response curves in relation to environmental exposure, Brouwer et al. (2017) suggested that only biologically plausible functions should be considered. Consequently, this was one of the criteria used in the present study when selecting the dose-response model used for baseline estimation of infection risk. Other criteria were that the endpoint should be infection; that the data used should originate from human disease outbreaks; and that the model should have already been used in other risk assessments. The dose-response model used for baseline estimation of infection risk was an exponential model including data from human disease outbreaks (Strachan et al., 2005) that has been used in other risk assessments (Franz et al., 2010; Boqvist et al., 2015; Brouwer et al., 2017). It takes the form:

(3) P=inf1-e∧[(-rd)]

where *r* = 1.13 × 10^–3^ (Strachan et al., 2005).

The exposure dose *d*, i.e., viable cells measured as CFU was modeled for 1 g of contaminated spinach with different degrees of damage to the leaves (undamaged, low, medium, and high) at 0, 1, and 2 dpi. The focus was on the relative change in risk in the different scenarios.

#### Calculation and Statistical Analysis

The experiment was based on a two-factorial design with four damage levels (factor 1) and three sampling events (dpi; factor 2). The results from viable counts were expressed as mean ± SD after log transformation (Angle et al., 1996). All data were analyzed using a general linear model (damage level; dpi; damage level^∗^dpi) followed by Tukey *B* test (*p* < 0.05) (Minitab vers 17.2.1; www.minitab.com). Further statistical analysis was performed in R studio (version 3.6.1.) (RStudioTeam, 2018) using the packages *ggplot2* for plotting and *ggpubr* for customization in ggplot2 plots. With the function *stat_summary()*, mean values were added to boxplots (indicated by red symbol within the box). *P*-values and significance levels were added to plots using the function *stat_compare_means()*, with one-way Anova used for comparing means for different damage levels. The function *geom_smooth()* was used to add regression lines to scatter plots. Coefficient of determination (R^2^) was calculated using the *stat_cor()* function.

## Results and Discussion

### Numbers of *E. coli* O157:H7 on Artificially Damaged Spinach Leaves

Non-washed spinach leaves retrieved from a commercial processing plant for leafy vegetables were used and the leaves were not decontaminated before starting the experiment, so they were inhabited by an autochthonous (*resident*) microbiome. Leaf weight and area were correlated, with leaf weight explaining 71% of leaf area variations (*p* < 0.001) (Figure 1B). Damaged leaf area varied widely between the damage treatments (Figure 1C) and separated the treatments into three distinct groups, i.e., with no (U), low (L), and moderate (M)/high (H) artificial damage. Thus, saturation in damaged leaf area was observed in the moderately damaged leaves, and additional leaf cuts did not significantly alter the damage level (Figure 1C). This might be because the proportion of large leaves was higher for group M than for the high damage group (Supplementary Figure 2), thus leading to more lesions per leaf (Figure 1D).

**FIGURE 2:**
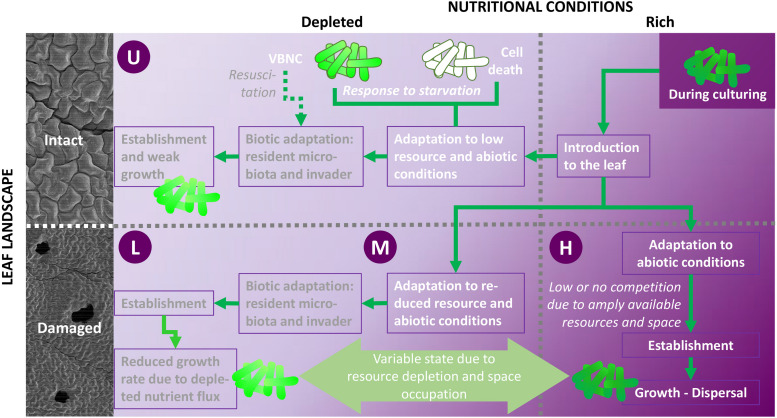
Study conditions and potential processes following inoculation of *E. coli* O157:H7 *gfp*+ on leaves with various damage levels (undamaged, U; low damage, L; moderate damage, M; high damage, H). Leaf injury (bruising) modifies both leaf landscape (removal of abiotic barriers, e.g., cuticle; 3D-extension of surface area to colonize, see also Figure 5, Supplementary Figures 5–7, and Supplementary Videos 1, 2) and the pool of readily available nutrients. The nutrient pulse exhibited through bruising (M) alleviates biotic competition for space and nutrients, allowing *E. coli* to maintain a well-functioning metabolism, to substantially grow (dark green cell shade), and to disperse. In comparison, leaf landscape and resource pool alterations are less pronounced in low- and medium-damaged leaves, leaving *E. coli* to cope to with varying degrees of abiotic and biotic hurdles, leading to reduced growth rates due to depleted nutrient flux (medium green cells). Resource availability in damaged leaves varies over time. Wounds may not be viewed as a static culture. The nutrient pulse due to high damage is most pronounced directly after wounding, leading to resource utilization and concomitant decreases in growth rates during days after wounding. Under low and moderate damage, cells may be excited by a secondary nutrient pulse due to decaying leaf tissue. This, together with resuscitation of metabolically compromised cells, allows high growth rates. Thus, growth response of *E. coli* to leaf damage is dependent on the resource phase. Nutrient scarcity (U, L) leads to cell death (open cells with dark green frame) or loss of culturability (viable but not culturable, VBNC; light green cells with dark green frame). Cells may resuscitate from the latter when nutrient availability increases. Thus, reluctant *E. coli* growth is expected under such conditions (Illustration: *B. Alsanius*; SEM photos: *E. Mulaosmanovic*).

The design of this study considered pathogen invasion with double perturbation dimensions caused by leaf damage, namely (i) nutrient pulse and (ii) landscape modification (Figure 2). Damaged leaves not only exhibit a richer pool of readily available nutrients, but also, at the damage site, a larger surface to be colonized, free from barriers existing in intact leaves. Different studies have investigated bacterial invasion on leaves (Melotto et al., 2006; Underwood et al., 2007; Barak et al., 2011; Van Elsas et al., 2012). With respect to plants, invasion as a concept has mainly been studied with respect to the microbial community structure of soils and roots (Kinnunen et al., 2016). Several studies have compared altered resource (nutrient) availability (Mallon, 2015; Mallon et al., 2015; Berg and Koskella, 2018), revealing contrasting invasion success. Although there is no such thing as an intact leaf (Mulaosmanovic et al., 2020) in natural or crop production and processing settings [Mulaosmanovic et al., submitted], the impact of modifications in leaf landscape and nutritional conditions can be assessed. The present study examined interactions between leaf damage size and invasion success of the target organism *E. coli* O157:H7, for which plants are not considered to be a primary habitat (Brandl, 2006), but did not quantify the strength of the nutrient pulse caused by wounding. Changes in the microbial community structure assisted by damage have previously been characterized by Mulaosmanovic et al. [submitted], and were not included in the present assessment of the biological and food safety relevance of leaf damage.

Although the inoculum density was standardized to log 6.74 ± 0.03 CFU × mL^–1^, the sum of culturable *E. coli* O157:H7 *gfp*+ × cm^–2^ detached from spinach leaves directly after inoculation varied between the four damage levels, with the highest numbers on moderately and severely damaged leaves, and the lowest on low damaged and undamaged leaves (M, H > U, L; Supplementary Table 1). This general trend was found throughout the experiment, and at first sight might appear to be a systematic experimental error. However, the inoculated cells were subjected to nutrient stress when transferred from the nutrient-rich propagation conditions to deprived circumstances in the inoculum suspension, and then to leaves with no or low damage. The difference in sum of *E. coli* attached to the leaves might then be a consequence of physiological changes due to starvation, leading to cell death or viable but not culturable (VBNC) cells (Nyström, 2001) and incapacity to overcome the abiotic and biotic hurdles of the leaf habitat. A limnological invasion study has found that inoculum density is a key factor for successful invasion (Acosta et al., 2015). The considerable load of *E. coli* O157:H7 does not compare with natural contamination conditions, but with the presence of a severe *E. coli* shedder (Omisakin et al., 2003). Comparable contamination rates were used in previous studies (Wright et al., 2017) and may occur when storing leafy vegetables at abuse temperature (Söderqvist et al., 2017). Furthermore, studies utilizing different inoculum densities show that inoculum size is not in itself the most decisive parameter for leaf colonization; populations with low densities at the start produce similar outcomes to *E. coli* populations with high initial densities (Wright et al., 2017). Crop type appears to be more crucial for the fate of *E. coli* on plants (Hartmann et al., 2017; Darlison et al., 2019).

### Impact of Leaf Damage on Leaf Surface Attachment by *E. coli* O157:H7

**FIGURE 3:**
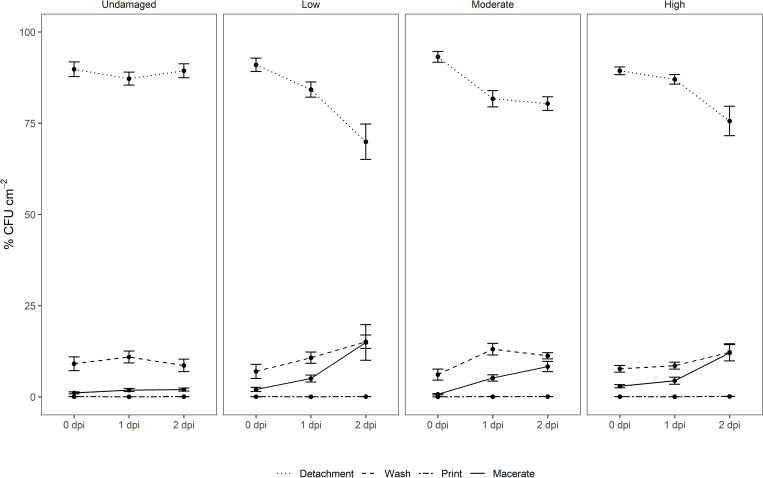
Relative distribution of *E. coli* O157:H7 *gfp*+ in suspensions after centrifugation (detachment, washing), after surface decontamination (leaf print), and in macerate of non-washed spinach leaves from a commercial leafy vegetable processing plant exposed to three levels of artificial damage (low, moderate, high) and leaves without intentional damage. Leaf analyses were performed directly after inoculation (0), and 1 and 2 days post-inoculation (dpi) of the target strain (*n* = 15).

Success of leaf surface attachment was monitored indirectly by analyzing the prevalence of *E. coli* O157:H7 *gfp*+ in the supernatant suspension after the repeated centrifugation (detachment, washing). The treatments used in this study to remove *E. coli* O157:H7 *gfp*+ loosely attached to spinach leaves directly after inoculation showed substantially higher removal rates than approaches using rinsing only (Uhlig et al., 2017) or immersion of leafy vegetables at various water temperatures and storage temperatures using *Listeria monocytogenes* (Natsou et al., 2012). Of the attached *E. coli* cells, 97.1–99.3% could be removed by the detachment and washing steps (M > U > L > H; % CFU × cm^–2^: 99.27A, 98.86A, 97.95A, 97.05A) directly after inoculation (Figure 3). This aligns with results presented by Jensen et al. (2015) and Pezzuto et al. (2016) when using high inoculum density of *Salmonella* spp. However, the proportion of cells removed from the total population through the two centrifugation (detachment, washing) steps decreased gradually over time in all treatments involving artificial leaf damage, leaving 2–15% of the total *E. coli* O157:H7 *gfp*+ associated with the leaves at 2 dpi (Figure 3). With respect to log values in the washed off fraction, the CFU of *E. coli* × cm^–2^ increased over time, indicating a greater total population (Supplementary Figure 3 and Supplementary Table 1). Proliferation and loose attachment of the inoculant, or resuscitation from VBNC, might serve as alternative explanations for this observation. The conflicting trends in removal rates of *E. coli* are puzzling. The lower removal rate of *E. coli* O157:H7 *gfp*+ from no and low damaged leaves might indicate better retention of *E. coli* O157:H7 *gfp*+ than on leaves with moderate and high damage. However, this was not supported by the results for the latter treatments. An alternative explanation could be that organic nutrients in the rinsate from moderately to highly damaged leaves might have supported reproduction of the inoculant. Interestingly, this effect persisted over time, whereas the initial nutrient influx due to damage should have been exhausted by 1 and 2 dpi. Rinsate nutrients cannot account for the increased number of *E. coli* O157:H7 *gfp*+ detached from moderately and highly damaged leaves directly after inoculation. Alternatively, wounding might impact the physiological status of *E. coli* O157:H7 *gfp*+ cells, retaining cells on highly wounded tissue in a viable and culturable stage, whereas cells on nutrient-depleted leaves progress to VBNC or die. Furthermore, wounding *per se* might create conditions more conducive to *E. coli* O157:H7 *gfp*+ cells passively adhering to the leaf surface, e.g., increased surface area, altered physical and chemical conditions, or altered net charge of leaf surface. The initial higher recovery is most likely a result of additional inoculation solution adhering the leaves due to change in the hydrophobicity of the leaf surface in damaged area. The total population of *E. coli* O157:H7 *gfp*+ undoubtedly grew more on damaged leaves. The numbers of introduced *E. coli* removed from moderately to severely damaged leaves at 1 and 2 dpi were higher, compared with leaves with no, or low levels of damage. We could speculate that leaves with damage nearing saturation partly supported greater population of *E. coli* over 24 and 48 h. This is based on the assumption that cells detach and wash off at similar rates, regardless of the level of damage inflicted on leaves. This, however, does not fully explain the magnitudes higher recovery after 1 and 2 dpi, and the explanations provided herein are not mutually exclusive.

### Impact of Leaf Damage on Internalized *E. coli* O157:H7

For surface sterilization, the commercially administered concentration of sodium hypochlorite was used. However, verification of the surface decontamination using abaxial and adaxial leaf printing on agar plates showed that the inoculated strain survived the treatment to a great extent as >90% of the leaf imprints were positive for growth of the artificially introduced strain. In fact, limited efficacy of standard sanitation practices for removal of *E. coli* has been reported in different studies (Allende et al., 2008, 2009; López-Gálvez et al., 2009; Ölmez, 2010; Keskinen and Annous, 2011; Davidson et al., 2013; Wright et al., 2017). A limitation of surface sterilization followed by maceration and plating is that bacteria which are not internalized, but somehow shielded from surface sanitation, may be classified as internalized. We therefore avoid referring to macerate-associated *E. coli* as “internalized,” as we could not fully exclude presence of shielded non-internalized bacterial cells on the leaf surface.

**FIGURE 4:**
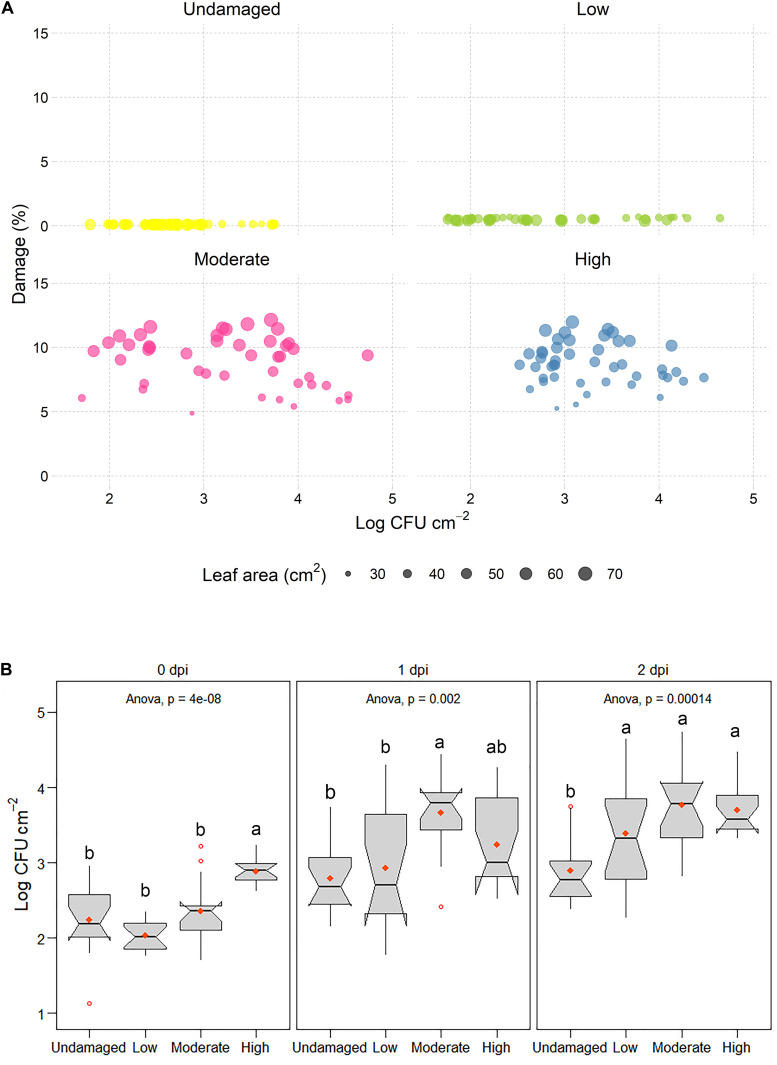
**(A)** Log CFU *E. coli* O157:H7 *gfp*+ × cm^– 2^ leaf area in leaf macerate as a function of predicted leaf damage (%). Circle size in scatter plots denotes the leaf area of *E. coli* O157:H7 *gfp*+ inoculated leaves. **(B)** Boxplots of log CFU *E. coli* O157:H7 *gfp*+ found in leaf macerate after 0, 1, and 2 dpi, calculated per cm^2^ of leaf area. Different letters above boxes indicate significant differences between damage levels (Tukey, *p* ≤ 0.05).

Determination of *E. coli* O157:H7 *gfp*+ concentrations in the macerate during incubation showed that overall log values varied significantly with respect to dpi (*p* < 0.001; Figure 3 and Supplementary Figure 3). Leaf damage had a significant impact on log CFU *E. coli* O157:H7 *gfp*+ re-isolated from the macerate (Figure 4A). Leaf weight, but not leaf area in all cases, was negatively correlated with the prevalence of *E. coli* counts in the macerate, and explained between 21 and 80% of the variation in the introduced organism. When exposed to the same level of damage, lighter leaves accumulated higher counts per gram tissue in the macerate. This may be due to leaf age rather than leaf weight (Supplementary Figure 4). By definition for commercially grown baby spinach leaves, younger baby spinach leaves are lighter. For iceberg lettuce, substantially richer nutritional conditions, and hence higher *E. coli* colonization, has been detected on younger than older leaves (Brandl and Amundson, 2008). This may indicate that young undamaged leaves pose a similar risk of causing food-borne disease outbreaks as young leaves with low or moderate damage.

**FIGURE 5:**
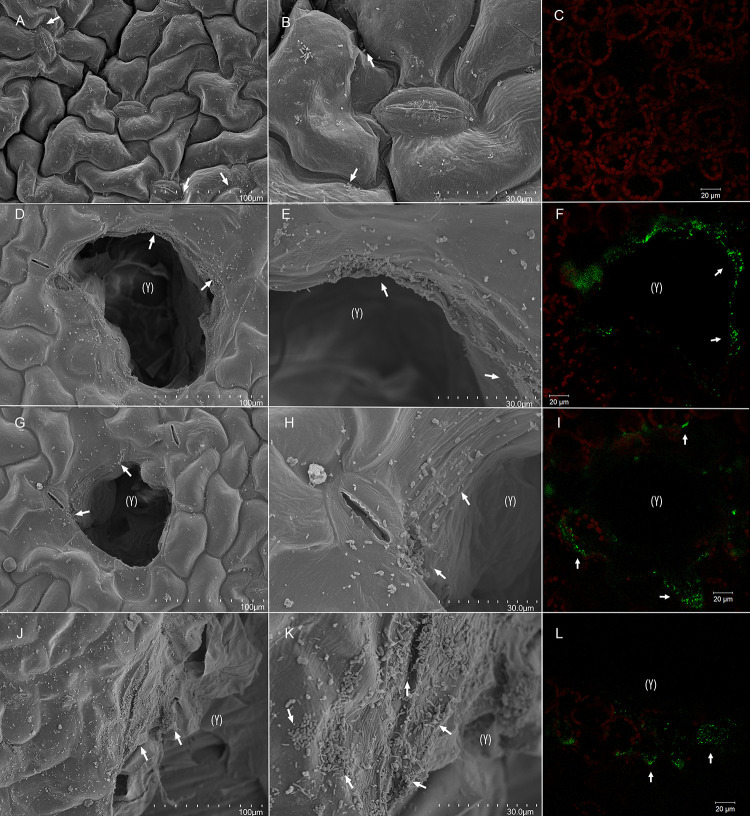
Bacterial colonization of artificially damaged spinach (*Spinacia oleracea* L.). Whole undamaged leaves **(A–C)**, and leaves with a low **(D–F)**, moderate **(G–I)**, and high **(J–L)** level of artificial damage were dip-inoculated with 10^6^ CFU × mL^– 1^ *E. coli* O157:H7 bacteria and observed 2 dpi. Leaves were surface-sanitized with 0.25% sodium hypochlorite for 20 s before sampling for confocal laser scanning microscopy (CLSM). From scanning electron micrographs (SEM) **(A,B,D,E,G,H,J,K)** and CLSM images **(C,F,I,L)**, it can be observed that lesion edges, junctions between adjacent epidermal cells, and stomata were the preferred colonization sites (arrows). Red fluorescence **(C,F,I,L)** indicates autofluorescence of the chlorophyll within chloroplasts, and green fluorescence indicates cells of *E. coli* O157:H7 bacteria. Lesions are marked with (Y). Scale bars 100 μm **(A,D,G,J)**, 30 μm **(B,E,H,K)**, and 20 μm **(C,F,I,L)**.

Interestingly, already directly after inoculation (0 dpi), the inoculated strain was significantly more prevalent in macerate of severely damaged leaves (H) than in macerate from the other three treatments (Figure 4B). This is particularly surprising as the removal rate of various washing and detachment steps was highest in the treatments with moderate and severe damage (Supplementary Figure 3 and Supplementary Table 1) and might indicate changes in leaf landscape (Figure 5) and environmental and nutritional conditions due to wounding (Figure 2). A gradual change in counts of the introduced strain occurred during the subsequent days. The introduced strain increased in all artificially damaged leaves, but the pace was slower in leaves with a lower damage level. The strongest increase in numbers of macerate-inhabiting *E. coli* was found within the first day post-inoculation, with significantly higher presence of *E. coli* in macerate of leaves with higher damage levels than lower levels (U, L). Nevertheless, *E. coli* counts were more than 36- and 20-fold higher in leaf macerate at damage level L and M, respectively. The increase in culturable *E. coli* O157:H7 *gfp*+ in macerate from non-damaged and severely damaged leaves was less dramatic (U: 4.3-fold increase; H: 5.3-fold increase). This might be a consequence of the nutritional limitations between the treatments, where *E. coli* O157:H7 *gfp*+ regained its culturability (Cuny et al., 2005), potentially following access to unexploited nutrient pulses in the L and M leaves, but not in undamaged leaves. After 48 h of incubation (2 dpi), no difference in occurrence of the inoculated *E. coli* strain (log CFU × cm^–2^) was found between leaves exposed to the three different levels of artificial damage. The increase in *E. coli* between dpi 1 and 2 was similar in macerate from damaged leaves (1.55- to 1.59-fold). The values all deviated significantly from log values of introduced *E. coli* in the non-damaged leaves, for which *E. coli* counts in leaf macerate increased 1.13-fold between dpi 1 and 2.

As the damaged area did not differ significantly between M and H leaves, a possible explanation for the higher amount of *E. coli* O157:H7 *gfp*+ associated with the macerate of highly damaged leaves directly after inoculation could be the type of damage. High damage introduces cuts to edges, changing a considerable part of the leaf to be hydrophilic rather than hydrophobic. This results in higher changes to the physicochemistry of the leaf, hence, more inoculum will adhere to the surface. Indeed, Takeuchi and Frank (2000) showed that *E. coli* O157:H7 preferably colonizes cut edges as opposed to the intact leaf surface, which is supported by the verification results obtained in the present study using confocal laser scanning microscopy (see section “Verification of Leaf Damage and *E. coli* O157:H7 Interactions Using Imaging”). By definition, leaf wounds are three-dimensional. In addition to the previously mentioned alteration in leaf landscape and abiotic conditions, the picture might be biased by the fact that quantification of damage area in this study was based on a two-dimensional assessment.

### Verification of Leaf Damage and *E. coli* O157:H7 Interactions Using Imaging

Although plants are not its primary habitat, *E. coli* O157:H7 is able to colonize leaves of leafy vegetables as both an epiphyte and endophyte (Deering et al., 2012; Hartmann et al., 2017; Wright et al., 2017). Microscopy-based techniques, such as scanning electron microscopy (SEM) and confocal laser scanning microscopy (CLSM) have been used to visualize plant-microbe interactions. Bacterial invasion into stomata or damaged tissue has been studied with SEM (Keskinen et al., 2009; López-Gálvez et al., 2010; Wang et al., 2010). Although SEM micrographs reveal presence of bacteria on leaf surface, with this approach it is not possible to distinguish between artificially introduced and residential bacteria. CLSM is the most common approach for corroborating presence of the particular fluorescent protein-tagged microorganism, and identifying internalized bacteria, where images can be taken at different microscopic depths. It has previously been used for visualization of internalized *E. coli* cells and colonies, and to determine depth of *E. coli* penetration into tissue of leafy vegetables (Takeuchi and Frank, 2000; Solomon et al., 2002; Brandl, 2008; Mitra et al., 2009; Hartmann et al., 2017; Wright et al., 2017).

Scanning electron micrographs of intact and artificially damaged and inoculated plant material at 2 dpi (Figure 5) revealed that bacteria on leaf surface preferred junctions between epidermal cells and stomatal openings on undamaged tissue. Edges of artificial lesions were highly colonized compared with undamaged leaf areas, and greater colonization was observed on larger wounds (Supplementary Video 2). CLSM assessment revealed green fluorescence along stomatal openings and in substomatal cavities on both intact and damaged leaves, even after surface sanitation with 0.25% sodium hypochlorite, but surface sanitation may have affected the density of *E. coli* O157:H7 *gfp*+ cells. CLSM scans of leaf lesions and adjacent areas at 2 dpi revealed increased surface colonization in the form of fluorescent matrix on the edges of artificial lesions and cells surrounding lesion (Figure 5 and Supplementary Figures 5–7). This indicates that, in addition to stomata, lesion edges may be preferred attachment sites, irrespective of their size, which corroborates previous findings (Takeuchi and Frank, 2000; Brandl, 2008).

Invasion of artificial wounds by *E. coli* O157:H7 *gfp*+ was confirmed in our study, and the severity of invasion was affected by the size of individual lesions (Figure 5, Supplementary Figures 5–7, and Supplementary Videos 1, 2), and relative leaf damage (Figure 4A). On leaves without artificial damage (U), *E. coli* O157:H7 *gfp*+ was mainly located in junctions between epidermal cells and along stomatal openings (Supplementary Figure 5B), or in sub-stomatal cavities at depth down to 5 μm (Supplementary Figure 5C and Supplementary Video 1). In artificially damaged leaves, invasion of the inoculant was observed in deeper tissue layers, 15–40 μm below the epidermal surface, with invasion depth depending on damage level (Supplementary Figures 5D–L). The greatest accumulation of *E. coli* O157:H7 *gfp*+ cells and penetration depth was observed with the high (H) damage level, next to the cut area, where *E. coli* O157:H7 *gfp*+ formed 8 μm × 8 μm sized colonies at a depth between 20 and 40 μm in the mesophyll tissue layer, possibly as a result of side internalization via artificial cut in the leaf tissue (Supplementary Figures 5J–L, 6J–L and Supplementary Video 2).

In lesions and adjacent areas in M and H damage leaves, aggregates of *E. coli* O157:H7 *gfp*+ were detected in the apoplast (Supplementary Figures 5, 7), a nutrient-rich habitat (Pignocchi and Foyer, 2003; Merget et al., 2019). *E. coli* O157:H7 *gfp*+ was found internalized in spaces between cells adjacent to lesions within the mesophyll tissue. This was characteristic for all damage levels, but particularly pronounced for leaves with high damage, where *E. coli* O157:H7 *gfp*+ was found at the similar depth in areas adjacent to lesions as in lesions (Supplementary Figures 7G–I). This indicates that, once internalized, *E. coli* O157:H7 *gfp*+ can multiply and disperse in apoplastic spaces, and not just persist as previously demonstrated (Deering et al., 2012; Wright et al., 2013). Bacterial invasion into the plant cells was not observed, only into the apoplastic spaces of the leaf and attached to mesophyll cells. This is in agreement with findings for *E. coli* Sakai (Wright et al., 2017) and for the same *E. coli* O157:H7 *gfp*+ strain used in the present study (Hartmann et al., 2017).

From the perspective of biological relevance, presence of leaf lesions and their size were found to affect the prevalence and invasion of *E. coli* O157:H7 *gfp*+ into leaf tissue of baby spinach. However, due to rapid proliferation after successfully breaching abiotic and biotic hurdles, the initial differences in CFU leveled out over the observation period at lower damage levels (L, M). As expected, driven by the suitable nutritional conditions, *E. coli* O157:H7 *gfp*+ preferentially colonized the edges of fresh wounds. Damage level played a role in depth of invasion and bacterial dispersal, with higher damage levels leading to deeper invasion and dispersal to adjacent areas. The lesion sizes tested were relevant for commercial settings. With respect to biological relevance of damage, new questions arise concerning e.g., the interactions between the persistently lower counts of *E. coli* O157:H7 *gfp*+ loosely associated with plant tissue and in macerate, and the presence and fate of VBNC cells. This study provided possible explanations for conflicting results, but we strongly recommend examination of this issue in forthcoming studies. From a consumer perspective, leaves displaying U-M damage levels would be macroscopically judged as immaculate in a shop display, while individual leaves exhibiting the most severe damage level (H) can occur in convenience leafy vegetable packages [Mulaosmanovic et al., submitted]. Mixing injured with macroscopically intact leaves inside the same bag enhances deterioration of intact leaves (Ariffin et al., 2017), which, in light of our results, may be of food safety relevance.

### Food Safety Risk Assessment

The starting point for the food safety risk assessment was a scenario with one contaminated leaf of spinach within a retail pack. In this study, contamination with the inoculation O157:H7 was considered. Although the leaves were washed and decontaminated, the inoculant internalized in some leaves. As a result of sodium hypochlorite treatment limitations, inoculated strain survived the sanitation treatment to a great extent (>90% positive leaf imprints). Therefore we acknowledge the presence of shielded non-internalized *E. coli* O157:H7 *gfp*+ cells on the leaf surface, retrieved from the macerate together with internalized cells. We considered the risk presented by a bag of spinach leaves (30 g or 70 g) with one or more internally contaminated leaves; whether the risk changed (increased) if the leaves were heavily damaged (macerate measured as undamaged, low, medium, and high); and whether the risk changed if leaves were included in the bag on the day of contamination or 1 or 2 days later.

Based on the geometrical means, the occurrence of culturable *E. coli* O157:H7 *gfp*+ in the macerate increased by one order of magnitude within 2 dpi. The risk of consumers’ infection from consumption of 1 g of contaminated spinach is shown in Table 1. The high likelihood of infection indicated by ingesting 1 g of leaves implies that even one contaminated leaf in a bag of 30 or 70 gram would present a high risk to the consumer.

**TABLE 1:** Risk of infection after consumption of 1 g of contaminated spinach.

*r* =	0.00113		
	**Days post-inoculation**		
**Level of damage**	0	1	2
Undamaged	0.999993	1	1
Low	0.9985	1	1
Medium	0.999999	1	1
High	1	1	1

Macroscopically intact leaves appeared to represent an infection risk if contaminated at high inoculum densities. This complements the post-EHEC 2011 outbreak debate (European Food Safety Authority [EFSA], 2011), and supports findings in earlier studies of Brandl and Mandrell (2002) for macroscopically intact leaf areas after co-inoculation of *Salmonella* Thompson and *Pantoea agglomerans*. It was demonstrated in this study for *E. coli* O157:H7 (Figures 3–5, Supplementary Figures 3, 5–7, Supplementary Video 1, and Supplementary Table 1).

Based on the risk assessment, the presence of one contaminated leaf in a 70 g pack appears to result in an infection risk of almost 100%, irrespective of leaf damage level. The infection risk remained high even 1 and 2 days after introduction of the inoculant. However, the inoculum density used in this study was very high. The inoculation parameters were chosen in order to retrieve the inoculant in the macerate even of undamaged leaves. It should be recalled that the inoculum density is comparable to the impact of exposure to fecal material from cattle, which are super-shedders (MacCabe et al., 2019). Moreover, the 15 s of submersion in inoculant was shorter than the usual exposure time in the primary washing step (Grudén et al., 2016).

The risk assessment findings extracted from this study are of interest for the establishment of hurdles and measures preventing transmission within the horticultural value network:

(i) The entire bag must be rejected if one contaminated leaf is present.
(ii) Wash water accidentally coming in contact with high loads of *E. coli* O157:H7 must be discarded at once, and the washing equipment must be cleaned and decontaminated adequately.

The incubation period used in the present experiment can be viewed as the interval between onset of contamination and consumption. To translate these results into prevention measures, two hurdles can be identified, namely:

(i) Interval between contamination and consumption
(ii) Damage level.

Each of these hurdles appear to represent around a one log reduction in bacterial numbers under high inoculum density conditions. Future studies on whether these hurdles represent the same log reduction at lower levels of contamination are needed.

The following food safety insights could be inferred from these results:

- Contamination earlier in the food chain may present a greater risk to the consumer than contamination later in the food chain.
- At high inoculum density, the contaminant load and risk are high in all leaf damage levels.

### Conclusion

This study showed that leaf damage has a significant impact on numbers of *E. coli* O157:H7 *gfp*+ retrieved from macerate. In order to follow the introduced strain in the different segments of plant tissue, we performed dip inoculation of entire single leaves. Due to the destructive nature of the analyses, the results indicate changes over time, but cannot be translated into growth and survival of the introduced strain. The results showed that:

(i) Adhesion of *E. coli* O157:H7 *gfp*+ on spinach leaf surface is enhanced with increasing lesion area and incubation time after inoculation.
(ii) Any size of leaf wound enables *E. coli* O157:H7 *gfp*+ to internalize into spinach leaf, but proliferation pattern varies depending on leaf damage area.
(iii) Macroscopically intact spinach leaves can allow internalization of *E. coli* O157:H7 *gfp*+.
(iv) Leaf integrity is fundamental to food safety prevention; at high inoculum densities, contamination is independent of leaf damage level.
(v) Damage level and interval between contamination and consumption can be used to suggest two new potential hurdles to enhance food safety.

## Data Availability Statement

The original contributions presented in the study are included in the article/Supplementary Material, further inquiries can be directed to the corresponding author.

## Author Contributions

EM and STW: conceptualization. EM, STW, and BWA: methodology. EM: validation, investigation, data curation, and visualization. EM, IV, and BWA: formal analysis. BWA: resources, project administration, and funding acquisition. BWA and EM: writing – original draft preparation. BWA, EM, STW, and IV: writing – review and editing; BWA and STW: supervision. All authors contributed to the article and approved the submitted version.

## Conflict of Interest

The authors declare that the research was conducted in the absence of any commercial or financial relationships that could be construed as a potential conflict of interest.

## Supplementary Material

Supplementary Figure 1 Methodology approach: Sampling, inoculation, and extraction of artificially added *E. coli* O157:H7 from individual spinach (*Spinacia oleracea* L.) leaves.

Supplementary Figure 2 Distribution of leaf area grouped by days post-inoculation (dpi) and level of damage. Leaf area of *E. coli* O157:H7 *gfp*+ inoculated leaves was measured in image analysis software ImageJ. Dashed horizontal line in the violin plots represents the overall mean across all the dpi and damage levels. Black dot within the violin indicates summary statistics (mean ± SD) within the damage level (*n* = 15). A GLM with interactions between damage levels and dpi, followed by Tukey test (*p* < 0.05). Letters above violins indicate which damage levels and dpi differ significantly in leaf area.

Supplementary Figure 3 Absolute abundance of artificially added *E. coli* O157:H7 *gfp*+ in suspensions after centrifugation (detachment, washing), after surface decontamination (leaf print), and in the macerate of spinach (*Spinacia oleracea* L.) leaves, exposed to three levels of artificial damage (low, moderate, high) and compared with leaves without intentional damage. Leaf analyses were performed directly after inoculation (0), and 1 and 2 days post-inoculation (dpi) of the target strain (*n* = 15).

Supplementary Figure 4 Correlation between leaf weight **(A)** and area **(B)**, and the prevalence of *E. coli* O157:H7 *gfp*+ counts re-isolated from leaf macerate.

Supplementary Figure 5 Colonization of artificially damaged spinach (*Spinacia oleracea* L.) leaves by *E. coli* O157:H7 bacteria labeled with GFP. Whole undamaged **(A–C)**, low **(D–F)**, moderate **(G–I)** and high **(J–L)** damaged leaves were dip-inoculated with 10^6^ CFU × mL^–1^ *E. coli* O157:H7 and observed at 2 dpi. Leaves were surface-sanitized with 0.25% sodium hypochlorite for 20 s before sampling for confocal laser scanning microscopy. Green *E. coli* O157:H7 channel **(A,D,G,J)**, red chlorophyll auto fluorescence channel **(B,E,H,K)**, and merged channels **(C,F,I,L)** are shown. Range of z-stacks 25–45 μm. Scale bars 20 μm.

Supplementary Figure 6 Colonization of artificially damaged spinach (*Spinacia oleracea* L.) leaves by *E. coli* O157:H7 bacteria labeled with GFP. Whole undamaged leaves **(A–C)**, and leaves with a low **(D–F)**, moderate **(G–I)**, and high **(J–L)** level of artificial damage were dip-inoculated with 10^6^ CFU × mL^–1^ *E. coli* O157:H7 and observed 2 dpi. Leaves were surface-sanitized with 0.25% sodium hypochlorite for 20 s before sampling for confocal laser scanning microscopy. Red fluorescence indicates autofluorescence of the chlorophyll within chloroplasts, and green fluorescence indicates cells of *E. coli* O157:H7 bacteria. Lesions are marked with (Y). From z-stack orthogonal projections **(A,D,G,J)**, maximum intensity projections of z-stacks **(B,E,H,K)**, and 3D z-stack projections **(C,F,I,L)**, it can be observed that lesion edges were the preferred colonization sites and entry points. Scale bars 20 μm.

Supplementary Figure 7 Colonization of tissue adjacent to lesions on artificially damaged spinach (*Spinacia oleracea* L.) leaves by *E. coli* O157:H7 bacteria labeled with GFP. Leaves with a low (**A–C)**, moderate **(D–F)**, and high **(G–I)** level of artificial damage were dip-inoculated with 10^6^ CFU × mL^–1^*E. coli* O157:H7 and observed at 2 dpi. For images of undamaged leaves, see Supplementary Figure 5 **A–C**. Leaves were surface-sanitized with 0.25% sodium hypochlorite for 20 s before sampling for confocal laser scanning microscopy (CLSM). Z-stack orthogonal projections of lesions and adjacent tissue of non-inoculated **(A,D,G,J)** and *E. coli* O157:H7-inoculated **(B,E,H,K)** samples, and 3D z-stack projections of inoculated plant material **(C,F,I,L)** are shown. Red fluorescence indicates autofluorescence of the chlorophyll within chloroplasts, and green fluorescence indicates cells of *E. coli* O157:H7 bacteria. Tissue adjacent to lesions is marked with (X) and lesions are marked with (Y). Range of z-stacks 25–35 μm. Scale bars 20 μm.

Supplementary Table 1 *E. coli* O157:H7 (log CFU × cm^–2^) re-isolated from wash water of spinach (*Spinacia oleracea* L.) leaves from two subsequent washing steps (detachment, washing).

Supplementary Video 1 3D rendering/animation of CLSM scans of undamaged spinach (*Spinacia oleracea* L.) leaves, inoculated with *E. coli* O157:H7 bacteria labeled with GFP and observed 2 days post-inoculation.

Supplementary Video 2 3D rendering/animation of CLSM scans of highly damaged spinach (*Spinacia oleracea* L.) leaves, inoculated with *E. coli* O157:H7 bacteria labeled with GFP and observed 2 days post-inoculation.
