# Supplementary material for: Size Matters: Biological and Food Safety Relevance of Leaf Damage for Colonization of Escherichia coli O157:H7 gfp+
Source: Front Microbiol. 2021 Jan 27;11:608086. doi: 10.3389/fmicb.2020.608086 (PMC7873480; doi:10.3389/fmicb.2020.608086)
Supplement: Supplementary Figure 1 — Methodology approach: Sampling, inoculation, and extraction of artificially added E. coli O157:H7 from individual spinach (Spinacia oleracea L.) leaves. [file Data_Sheet_1.zip › Table S1.docx]

**Supplement Table 1.** *E. coli*O157:H7 *gfp+* (Log CFU cm^-2^) reisolated from wash water of spinach (*Spinacia oleacera* L.) leaves from two subsequent washing steps (detachment, washing). The spinach leaves were subjected to different levels of artificial damage (undamaged, low, moderate, high) before dip inoculation with the target strain. Samples were taken directly after damage and after 1 and 2 days post inoculation (dpi).

|  | **Detachment (Step 1)** | | | **Washing (Step 2)** | | |
| --- | --- | --- | --- | --- | --- | --- |
| **Damage level** | **0 dpi** | **1 dpi** | **2 dpi** | **0 dpi** | **1 dpi** | **2 dpi** |
| **Undamaged** | 4.4 D^1^ | 4.6 ABC | 4.6 ABC | 3.2 GH | 3.6 EFG | 3.5 FGH |
| **Low** | 4.0 DE | 4.3 CD | 4.3 CD | 2.7 I | 3.3 FGH | 3.5 FGH |
| **Moderate** | 4.7 ABC | 4.9 A | 4.9 AB | 3.1 HI | 4.1 DE | 4.0 DE |
| **High** | 4.5 ABC | 4.7 ABC | 4.6 ABC | 3.3 FGH | 3.6 EFG | 3.7 EF |

^1^ Values followed by different labels within the same washing step are significantly different with respect to GLM followed by Fisher’s test (p<0.05).
