# Supplementary figures and images for: Size Matters: Biological and Food Safety Relevance of Leaf Damage for Colonization of Escherichia coli O157:H7 gfp+
Source: Front Microbiol. 2021 Jan 27;11:608086. doi: 10.3389/fmicb.2020.608086 (PMC7873480; doi:10.3389/fmicb.2020.608086)

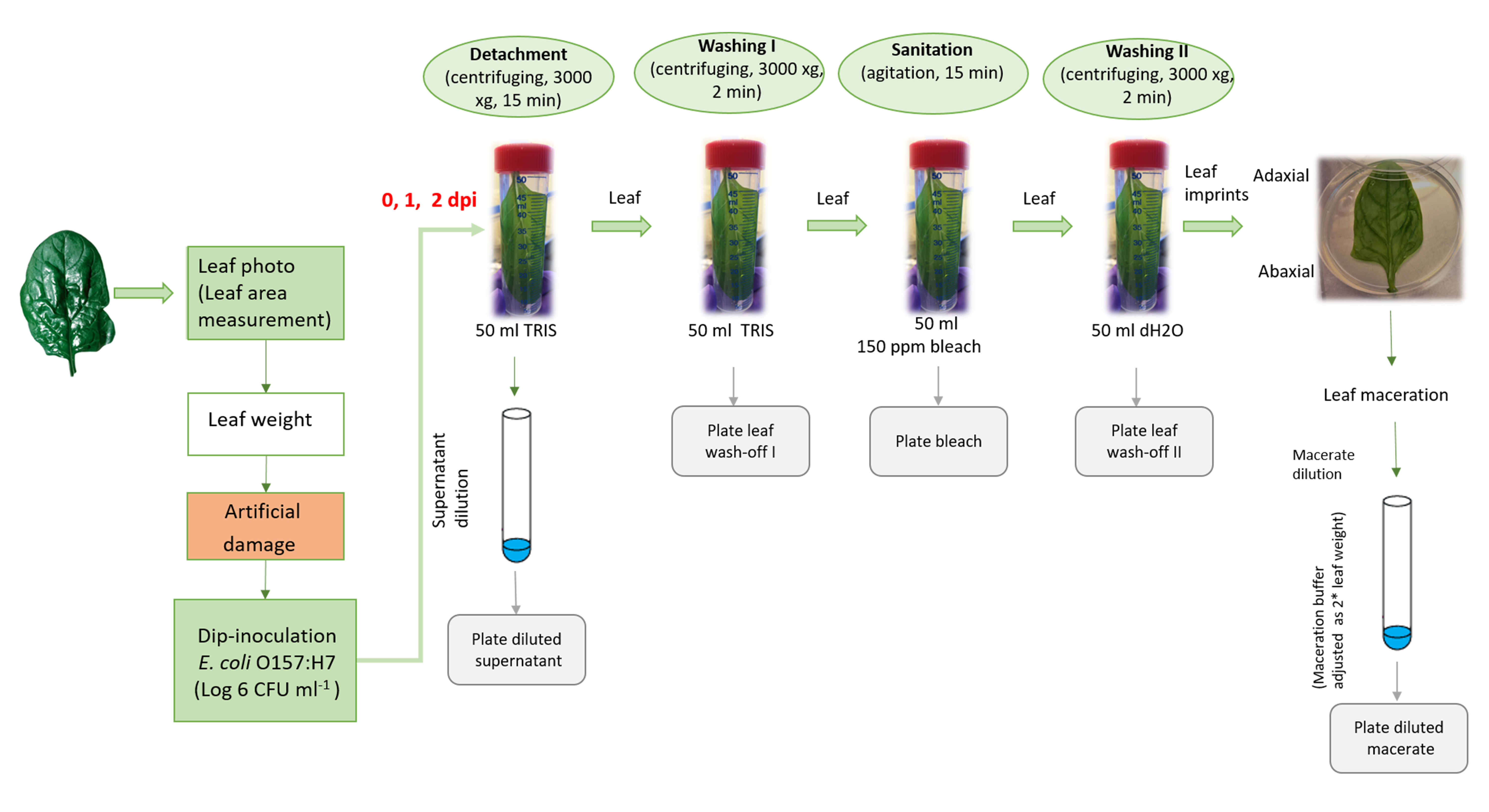

Supplement: Supplementary Figure 1 — Methodology approach: Sampling, inoculation, and extraction of artificially added E. coli O157:H7 from individual spinach (Spinacia oleracea L.) leaves. [file Data_Sheet_1.zip › Figure S1.png]

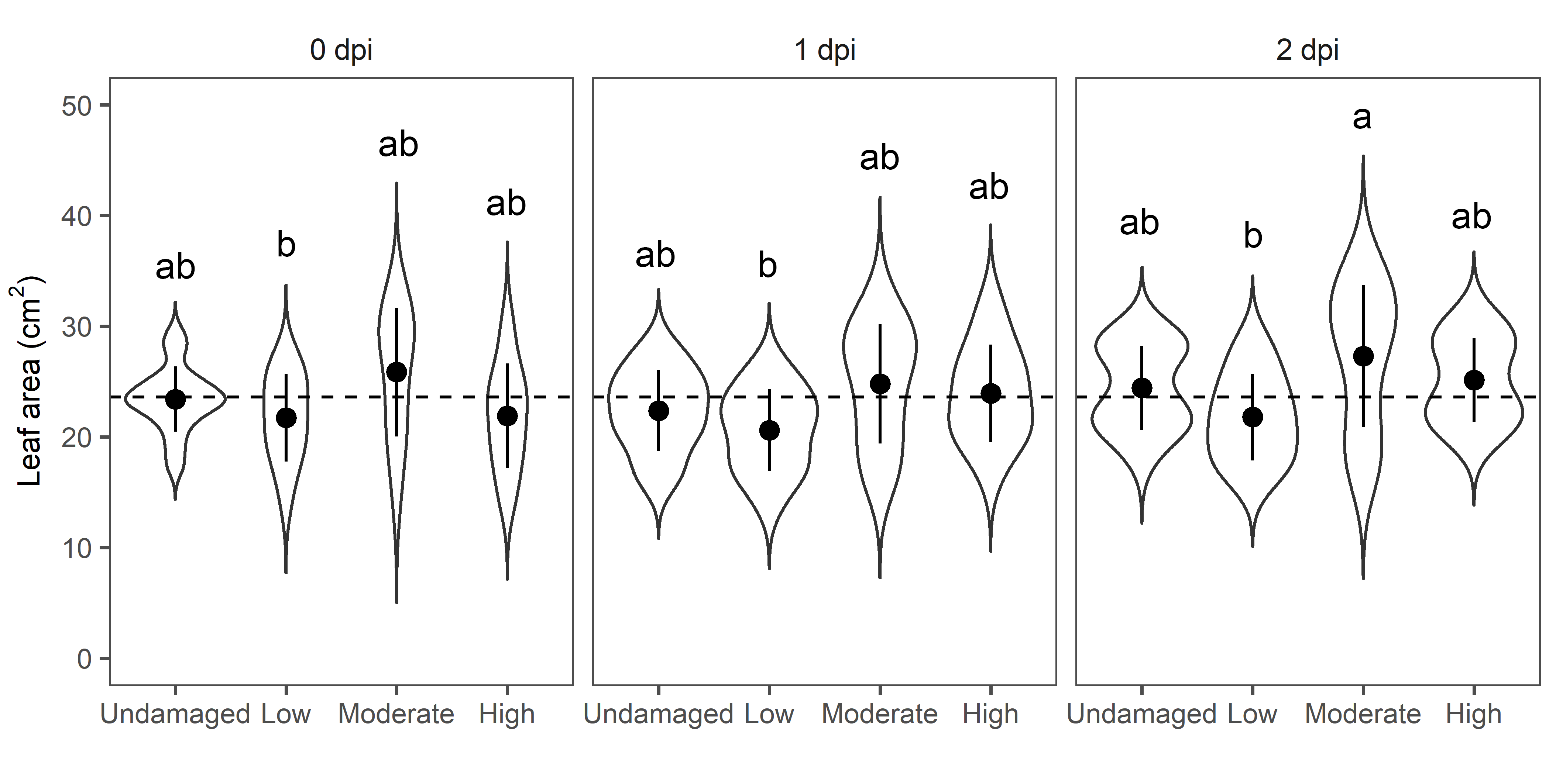

Supplement: Supplementary Figure 1 — Methodology approach: Sampling, inoculation, and extraction of artificially added E. coli O157:H7 from individual spinach (Spinacia oleracea L.) leaves. [file Data_Sheet_1.zip › Figure S2.png]

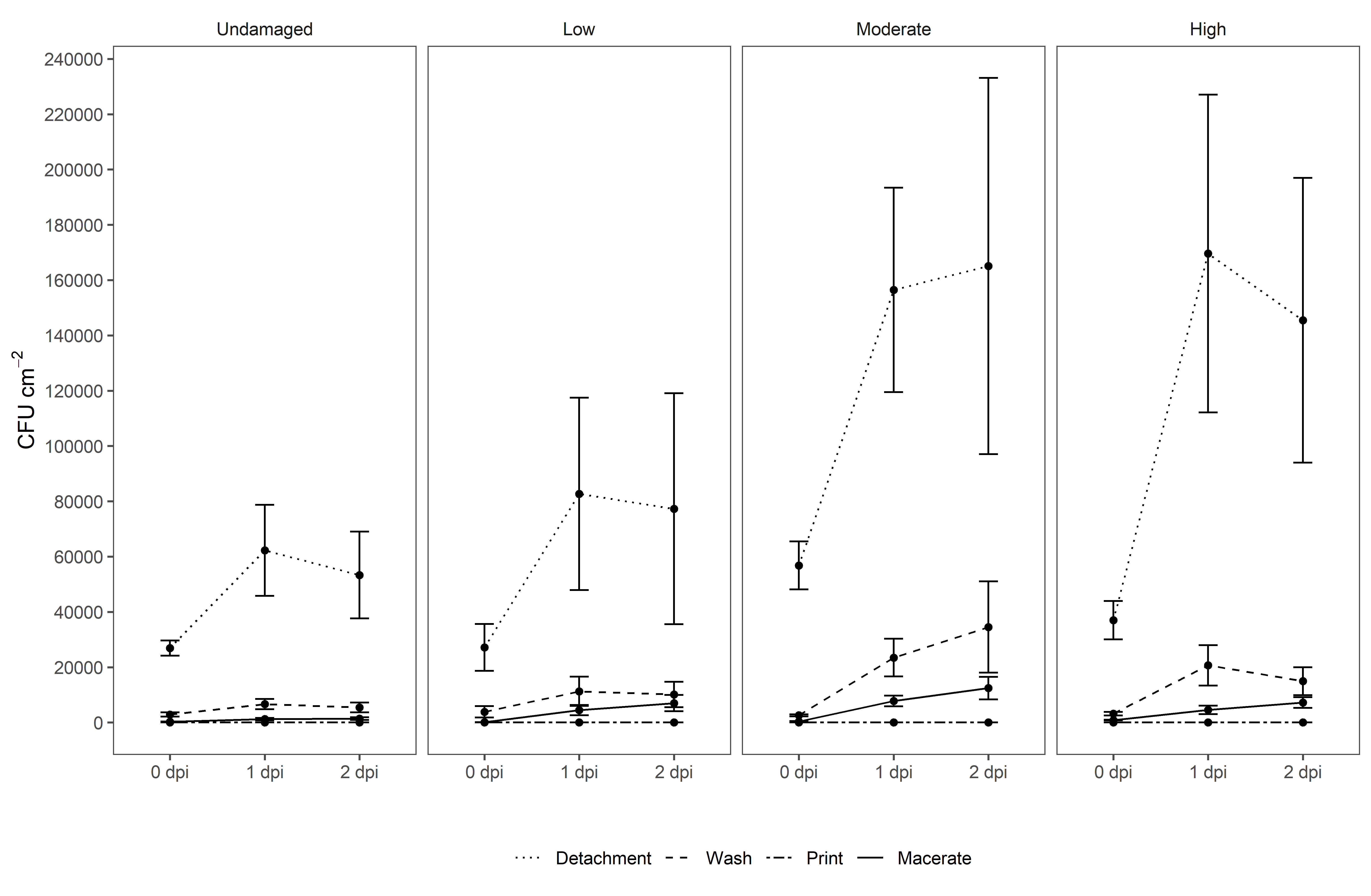

Supplement: Supplementary Figure 1 — Methodology approach: Sampling, inoculation, and extraction of artificially added E. coli O157:H7 from individual spinach (Spinacia oleracea L.) leaves. [file Data_Sheet_1.zip › Figure S3.jpg]

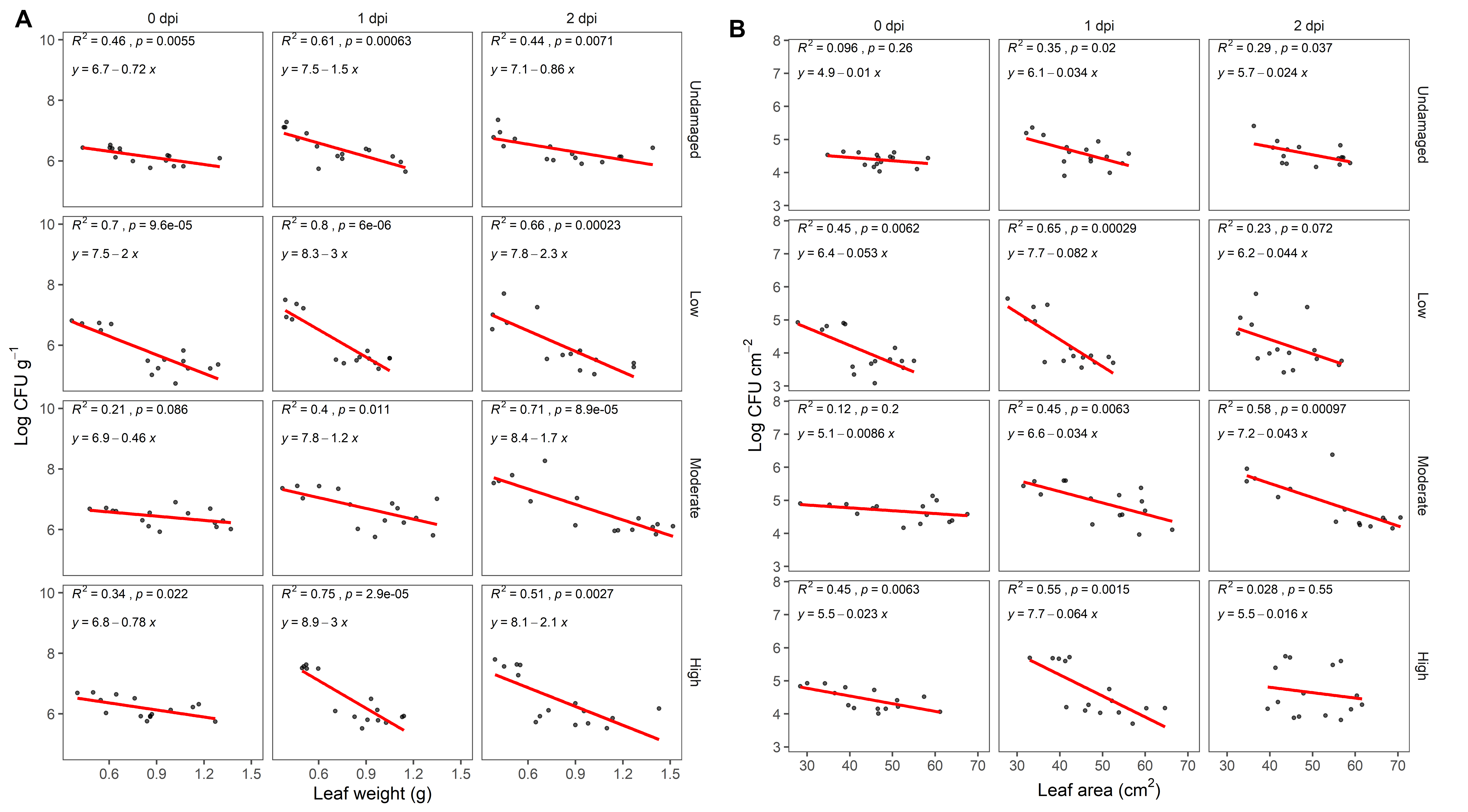

Supplement: Supplementary Figure 1 — Methodology approach: Sampling, inoculation, and extraction of artificially added E. coli O157:H7 from individual spinach (Spinacia oleracea L.) leaves. [file Data_Sheet_1.zip › Figure S4.png]

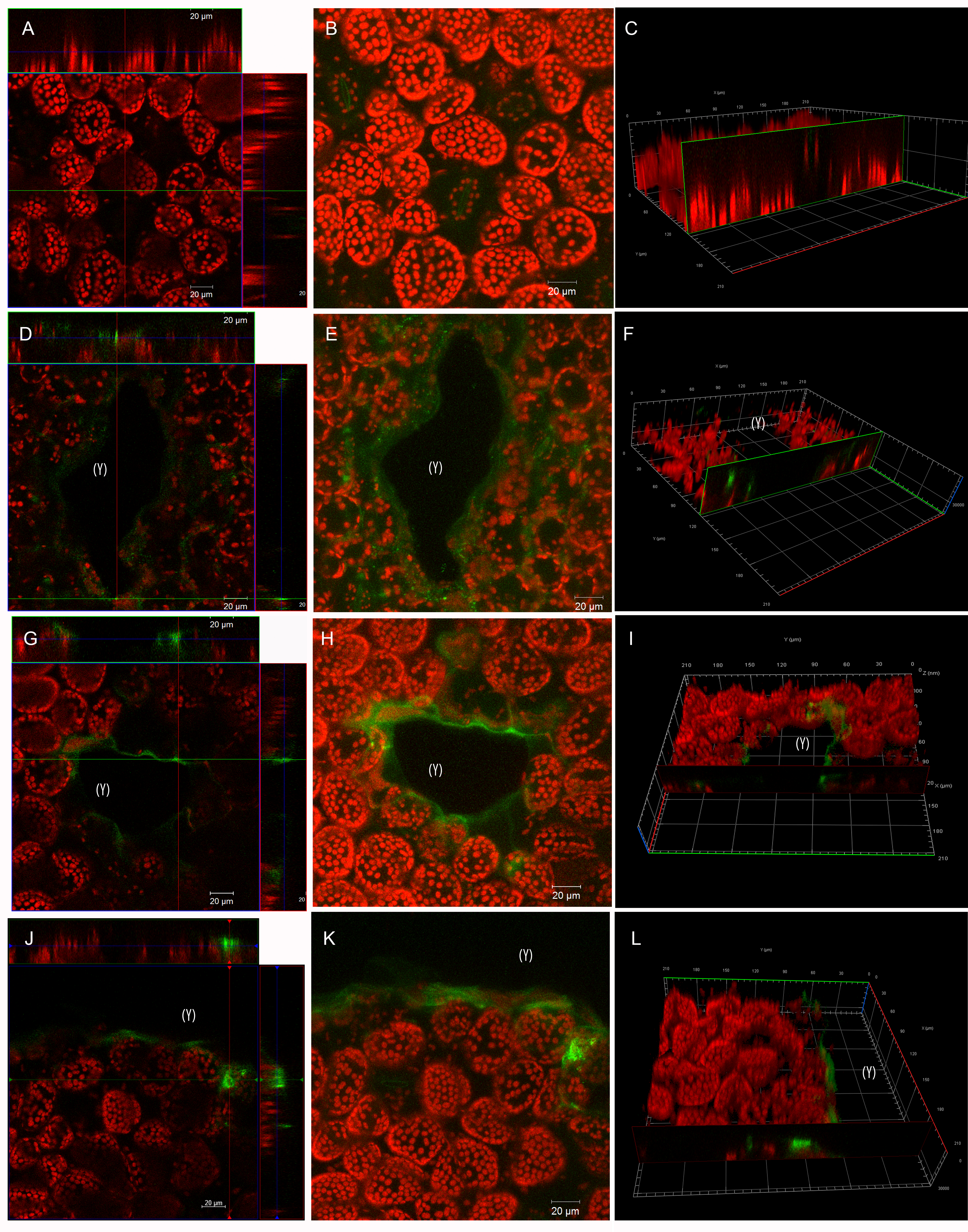

Supplement: Supplementary Figure 1 — Methodology approach: Sampling, inoculation, and extraction of artificially added E. coli O157:H7 from individual spinach (Spinacia oleracea L.) leaves. [file Data_Sheet_1.zip › Figure S5.jpg]

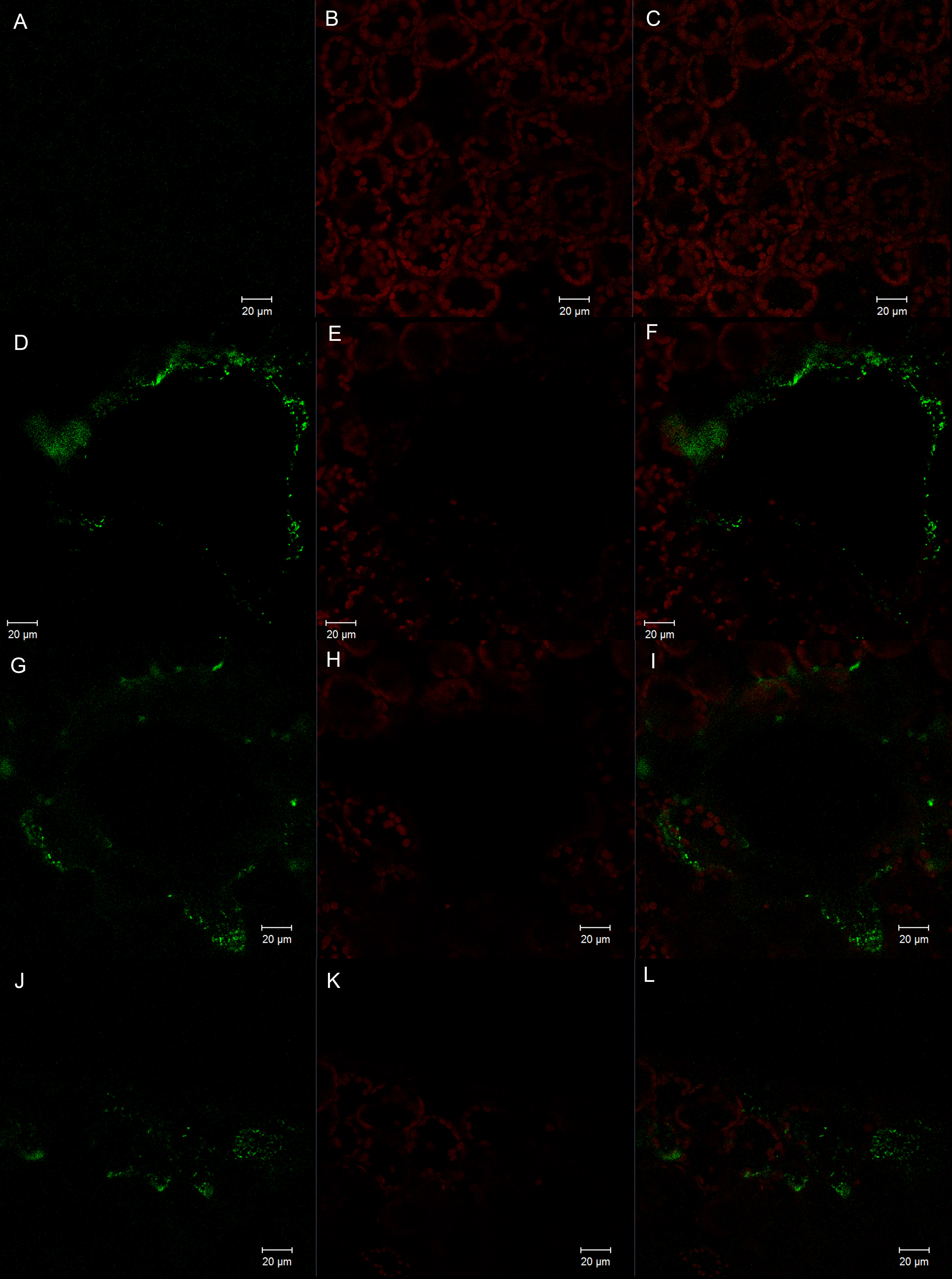

Supplement: Supplementary Figure 1 — Methodology approach: Sampling, inoculation, and extraction of artificially added E. coli O157:H7 from individual spinach (Spinacia oleracea L.) leaves. [file Data_Sheet_1.zip › Figure S6.jpg]

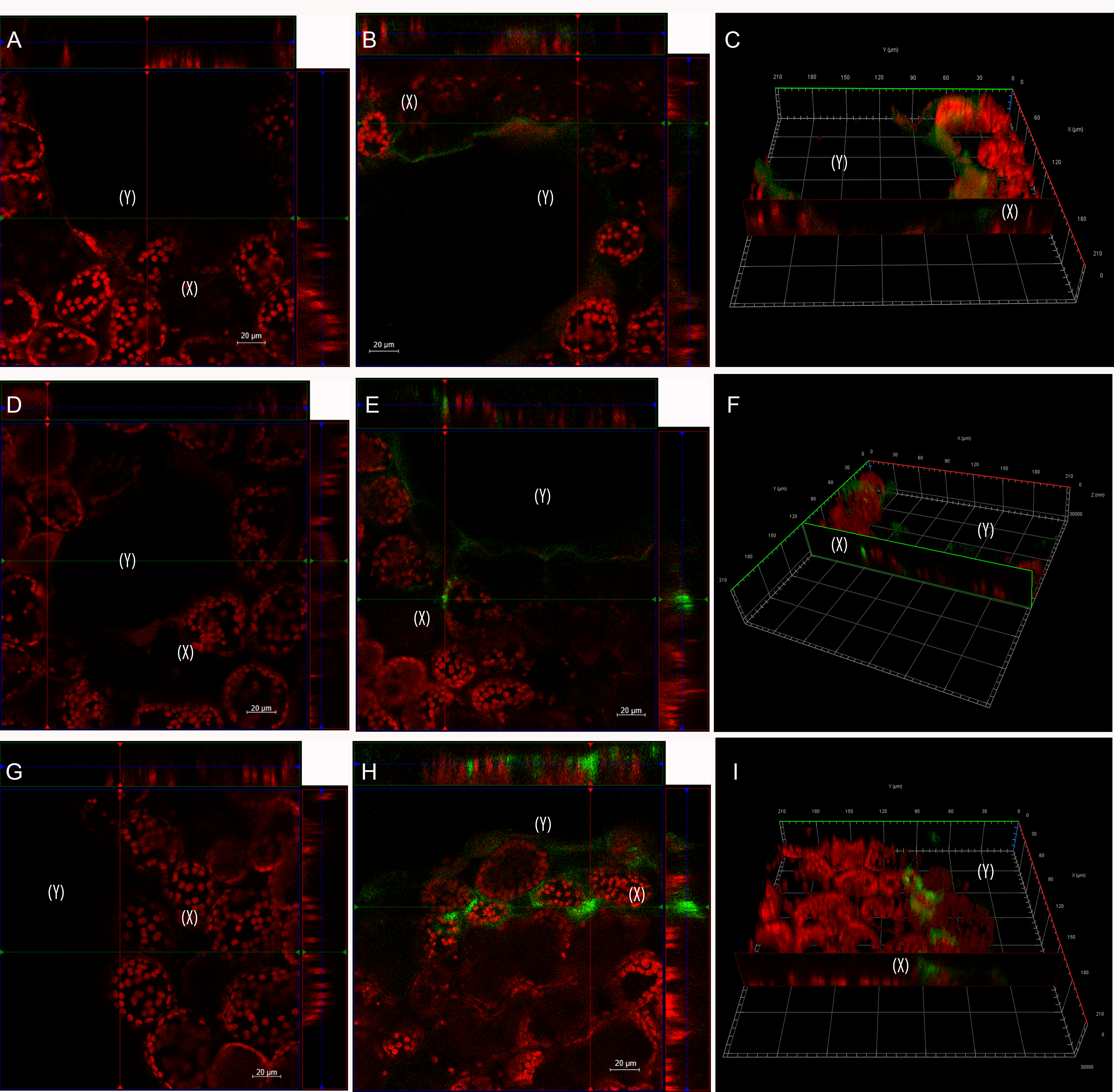

Supplement: Supplementary Figure 1 — Methodology approach: Sampling, inoculation, and extraction of artificially added E. coli O157:H7 from individual spinach (Spinacia oleracea L.) leaves. [file Data_Sheet_1.zip › Figure S7.jpg]
